# Long‐term effects of gamma frequency sensory stimulation after 30 months in patients with mild Alzheimer’s disease: Evidence supporting potential disease modification

**DOI:** 10.1002/alz70859_107168

**Published:** 2025-12-26

**Authors:** Diane Chan, Brennan L Jackson, Gabrielle de Weck, Kenji Aoki, Ute Geigenmuller, MJ Quay, Monica Zheng, Andrew Becker, Erika Ruiz, Remi Philips, Noah Milman, Elizabeth B Klerman, Vanesa Fernandez Avalos, Erin Kitchener, Michael Brickhouse, Alexandra Touroutoglou, Brad C. Dickerson, Edward S Boyden, Emery N Brown, Li‐Huei Tsai

**Affiliations:** ^1^ Massachusetts General Hospital, Boston, MA USA; ^2^ Massachusetts Institute of Technology, Cambridge, MA USA; ^3^ Harvard University, Cambridge, MA USA; ^4^ John A. Burns School of Medicine, Honolulu, HI USA; ^5^ Oregon Health & Science University, Portland, OR USA; ^6^ UCSD, San Diego, CA USA; ^7^ William James College, Newton, MA USA; ^8^ Massachusetts General Hospital and Harvard Medical School, Boston, MA USA; ^9^ Howard Hughes Medical Institute, Chevy Chase, MD USA; ^10^ Broad Institute of MIT and Harvard, Cambridge, MA USA; ^11^ Picower Institute for Learning and Memory, Cambridge, MA USA

## Abstract

**Background:**

Non‐invasive gamma‐frequency light and sound stimulation at 40Hz has been shown to reduce Alzheimer’s disease (AD) pathology and enhance behavioral performance in AD mouse models. Building on these findings, we hypothesized that induced gamma neural activity through light and sound stimulation could serve as a disease‐modifying therapy for AD. In the open‐label extension phase of this trial, our objective is to assess the effects of long‐term daily 40Hz stimulation.

**Methods:**

We conducted a placebo‐controlled, randomized control trial in subjects with probable mild AD to use our light and sound device at home for one hour daily (NCT 04042922). In the open‐label extension phase, we report results after 30 months of daily 40Hz synchronized light and sound in late‐onset AD (n=3) and early‐onset AD (n=2) patients. Longitudinal electroencephalogram (EEG) was used to evaluate for safety and neural activity when using the 40Hz stimulation. Magnetic resonance imaging (MRI) was used to evaluate brain structure and actigraphy was used to record sleep. Longitudinal neuropsychiatric testing and actigraphy were used to assess cognition and sleep. Plasma pTau217 levels were evaluated longitudinally.

**Results:**

Chronic 40Hz light and sound stimulation was safe and well‐tolerated after 30 months of daily usage. EEG data show that our novel light and sound device continue to safely and effectively induce 40Hz neural activity. Daily 40Hz stimulation slowed brain atrophy as compared to historical age‐matched controls. There was improvement of intradaily stability in 3 out of the 5 participants. Performance on cognitive testing scores improved in late‐onset AD patients but not as much in early‐onset AD patients. There was a reduction of plasma pTau217 by 47% in 1 participant as compared to baseline.

**Conclusions:**

Gamma frequency light and sound stimulation can be safely used daily for 30 months and slows AD‐related degeneration. Induced neural activity using sensory stimulation at 40Hz shows promise as a novel disease modifying therapeutic for Alzheimer’s dementia.

